# Cutaneous impact location: a new tool to predict intracranial lesion among the elderly with mild traumatic brain injury?

**DOI:** 10.1186/s13049-020-00781-2

**Published:** 2020-08-31

**Authors:** Xavier Dubucs, Frederic Balen, Eric Schmidt, Mathieu Houles, Sandrine Charpentier, Charles-Henri Houze-Cerfon, Dominique Lauque

**Affiliations:** 1 Emergency Department, Centre Hospitalo-Universitaire de Toulouse, Toulouse, France; 2 Department of Neurosurgery, Centre Hospitalo-Universitaire de Toulouse, Toulouse, France; 3 Department of Geriatric Medecine, Centre Hospitalo-Universitaire de Toulouse, Toulouse, France

**Keywords:** Elderly, Mild traumatic brain injury, Epidemiology, Emergency department

## Abstract

**Background:**

Mild traumatic brain injury is the leading cause of arrivals to emergency department due to trauma in the 65-year-old population and over. Recent studies conducted in ED suggested a low intracranial lesion prevalence. The objectives of this study were to assess the prevalence and risk factors of intracranial lesion in older patients admitted to emergency department for mild traumatic brain injury by reporting in the emergency department the precise anamnesis of injury and clinical findings.

**Methods:**

Patients of 65 years old and over admitted in emergency department were prospectively included in this monocentric study. The primary outcome was the prevalence of intracranial lesion threw neuroimaging.

**Results:**

Between January and June 2019, 365 patients were included and 66.8% were women. Mean age was 86.5 years old (SD = 8.5). Ground-level fall was the most common cause of mild traumatic brain injury and occurred in 335 patients (91.8%). Overall, 26 out of 365 (7.2%) patients had an intracranial lesion. Compared with cutaneous frontal impact (medium risk group), the relative risk of intracranial lesion was 2.54 (95% CI 1.20 to 5.42) for patients with temporoparietal or occipital impact (high risk group) and 0.12 (95% CI 0.01 to 0.93) for patients with facial impact or no cutaneous impact (low risk group). There was not statistical increase in risk of intracranial injury with patients receiving antiplatelets (RR = 1.43; 95% CI 0.68 to 2.99) or anticoagulants (RR = 0.98; 95% CI 0.45 to 2.14).

**Conclusion:**

Among patients of 65 years old and over, the prevalence of intracranial lesion after a mild traumatic brain injury was similar to the younger adult population. The cutaneous impact location on clinical examination at the emergency department may identify older patients with low, medium and high risk for intracranial lesion.

## Introduction

Mild traumatic brain injury (mTBI) is the leading cause of arrivals to emergency department (ED) due to trauma in the 65-year-old population and over [1, 2]. Lately, elderly visits to ED for mTBI have increased disproportionately, mTBI in the elderly is associated with an increase in morbidity and mortality. This is a frequent reason for hospitalization and it is associated with an alteration in functional and cognitive capacities [2, 3]. Current international guidelines are consistent with the large indication of non-contrast head computed tomography scan (head CT-scan) after mTBI in patients over 65 years old, even without initial consciousness loss [4, 5]. Head CT-scan is also recommended in all patients under antiplatelets or anticoagulants after mTBI. Former cohorts from which most international guidelines were derived showed that the intracranial lesions prevalence after mTBI in the elderly was higher than in younger subjects. Regarding to this population, intracranial lesion increased until 30% [6]. These guidelines are also discussed with specific elderly epidemiological and physiological features. Older patients showing less specific sign of intracranial lesion following head trauma compared to younger patients, they also present less of signs of intracranial hypertension. As well, the Glasgow score is less sensitive and the event taking place during the mTBI is often missing [7, 8].

Recent studies conducted in ED suggested a low intracranial lesion prevalence reaching 2.2% and a rate of neurosurgery lower than 1% [9]. These data suggest that the brain CT-scan indication following mTBI in this population could be more targeted. In addition to the cost issue, head CT-scan overuse could also have an impact on the patient in terms of radiation-induced neoplasia and cataracts [10]. A prospective study containing detailed injury history and clinical findings at the ED is required to better characterize prevalence and potential risk factors of intracranial lesions.

Our objectives were to assess the prevalence of mTBI-related intracranial lesions in subjects over 65 years old admitted to the ED and to identify risk factors for intracranial lesions by recording precise mTBI anamnesis as well as clinical findings.

## Method

### Study design

We conducted a prospective descriptive observational study in the two urban University Hospital EDs. These two EDs treated 78,000 and 40,000 patients in 2018 respectively, they located in the middle of a health care pool of approximately 1 million inhabitants.

Between January 2019 and June 2019, all consecutively patients aged 65 years old and older with mTBI admitted to the ED were included before head CT-scan. mTBI was defined as a traumatic brain injury with Glasgow score of 13 or higher on arrival in the ED. Decision to perform head CT-scan was made by the treating physician according to current national guidelines [11]. Patients without head CT-scan performed within the first 24 h after ED entry were excluded.

Based on the standardized questionnaire filled out in the ED, different types of residence place were reported: living alone at home, with relatives at home (defined by the presence of at least one relative living at home, e.g. wife; child/children) or in a nursing home (NH)). Antiplatelet (aspirin, clopidogrel), anticoagulants (warfarin, direct oral anticoagulant (Xa/IIa inhibitors) or subcutaneous anticoagulant) and psychoactive drugs were also recorded (benzodiazepine, antidepressant, neuroleptic, antiepileptic). History of neurosurgical intervention and cognitive impairment were included. The patient’s frailty was assessed by the *Clinical Frailty Scale* from 1 (very fit) to 7 (fully functionally dependent) [12]. mTBI was described by the time of the event, witness presence and the injury kinetic (ground-level fall, > 1 m or 5 steps, road accident, head striking by an object). In case of fall event, the precipitating factor was described: mechanical, faintness or vertigo, syncope. The transient symptoms after the injury were also reported (loss of consciousness, vomiting, seizure, amnesia, headache) and activity after falling (getting up alone or with assistance, staying on floor > 1 h). Unavailable variables after patient, witness, or NH requests were categorized as “unknown”. Glasgow score, focal neurological signs, basal skull fracture signs (otorrhagia, otorrhea, bilateral periorbital ecchymosis) and cutaneous injury types (cutaenous abrasion, hematoma, wound requiring suture, no cutaneous lesion) were recorded by the treating physician. He also showed the cutaneous impact location on a head figure. The cutaneous impact location was then categorized by the investigator as follow: frontal, temporoparietal, occipital, facial, or no cutaneous impact. After the ED visit, the patient’s outcome was noticed: discharge or hospitalization. According to ED and hospitalization reports, traumatic injury and/or medical emergency associated with the mTBI were described as well. Traumatic injuries were categorized as facial and/or peripheral fractures (spine, limbs), moreover medical emergency associated with mTBI included infectious conditions (pneumopathy, urinary tract infection), rhabdomyolysis, renal failure, and/or a post-fall syndrome. In the absence of any traumatic or medical emergency and/or wound requiring suture, mTBI was classified as isolated-mTBI. Additionally, any alcohol intoxication was included in g/l.

### Outcome measure

The primary outcome was an intracranial lesion found on a head CT-scan. As part of routine care, all head CT-scans were interpreted by a senior neuro-radiologist who provided a written report. Intracranial lesions were described as follow: subarachnoid hemorrhages, acute subdural, intra-parenchymal hematoma and/or cerebral contusion. Cortical subcortical atrophy was also notified according to the neuro-radiologist’s report.

### Sample size

The estimated number of patients required with a 95% confidence interval with a 10% width was 365 patients, based on an hypothesis of 5% intracranial lesion prevalence [4, 9].

### Statistical analysis

The intracranial lesion prevalence was described by frequency. Quantitative data were reported as an average with standard deviation (SD) or median with interquartile range (IQR) when the distribution was not normal. In univariate analysis, patients with and without intracranial lesion were compared with the Student’s t-test for quantitative data and the Chi-squared test or the Fisher’s exact test for qualitative data according to their respective conditions of use. Differences were considered significant if *p* <  0.05. Relative risks with 95% confidence interval were calculated for significant variables. Due to the sample size, a multivariate analysis was not performed. Statistical tests were conducted with Stata v11.2.

## Results

Between January 2019 and June 2019, 365 patients were included. The average age of the entire cohort was 86.5(±8.5) years old, ranging from 65 to 104, and 244 patients (66.8%) were female. Overall, 141 patients (38.6%) were receiving antiplatelet and 128 (35.1%) anticoagulant medication. One hundred and twenty-six patients (34.5%) presented cognitive impairment prior to ED visit.

The most common mTBI mechanism was ground-level fall (331/365, 91.8%). Among the 7 patients (1.9%) who had a road accident, 3 were drivers or passengers of a motor vehicle, 3 were cyclists and one was a pedestrian hit by a car. The mean blood alcohol level of the 7 inebriated patients was 2.2 g/l (SD = 1.1).

Furthers characteristic of the study population with univariate analysis for intracranial lesions are presented in Table 1.

**Table 1:** Characteristics of patients presenting to the ED with mild traumatic brain injury and univariate analysis according to the presence of intracranial lesion

	Population (***N*** = 365)	Intracranial lesion (***N*** = 26)	No intracranial lesion (***N*** = 339)	***p***-value
Age (years, SD)	86.5 (8.5)	86.8 (8.6)	86.4 (8.5)	0.80
Age < 75 (n, %)	41 (11.2)	2 (7.7)	39 (11.5)	
Age ≥ 75 (n, %)	324 (88.8)	24 (92.3)	300 (88.5)	0.55
Gender, female (n, %)	244 (66.8)	22 (84.6)	222 (65.5)	0.05
**Residence (n, %)**				
Nursing home	144 (39.5)	10 (38.5)	134 (39.5)	0.92
Home, with relatives	115 (31.5)	9 (34.6)	106 (31.3)	0.72
Home, alone	106 (29.0)	7 (26.9)	99 (29.2)	0.81
**Antiplatelets (n, %)**				
Aspirin	112 (30.7)	11 (42.3)	101 (29.8)	0.18
Clopidogrel	27 (7.4)	1 (3.8)	26 (7.7)	0.40
Aspirin + Clopidogrel	2 (0.55)	0 (0)	2 (0.59)	0.86
**Anticoagulant (n, %)**				
Warfarin	60 (16.4)	5 (19.2)	55 (16.2)	0.69
Direct oral anticoagulant	61 (16.7)	3 (11.5)	58 (17.1)	0.34
Subcutaneous anticoagulant	7 (1.9)	1 (3.8)	6 (1.8)	0.40
**Psychoactive drug (n, %)**				
Benzodiazepine	100 (27.4)	10 (38.4)	90 (26.5)	0.19
Antidepressant	65 (17.8)	8 (30.8)	57 (16.8)	0.07
Neuroleptic	18 (4.9)	0 (0.0)	18 (5.3)	0.25
Antiepileptic	11 (3.0)	0 (0.0)	11 (3.2)	0.43
**History**				
Cognitive impairment (n, %)	126 (34.5)	9 (34.6)	117 (34.5)	0.99
Clinical Frailty Scale (mean, SD)	4.6 (1.6)	4.9 (1.7)	4.6 (1.6)	0.33
Neurosurgical intervention	10 (2.7)	0 (0.0)	10 (2.9)	0.47

Median time between mTBI and ED arrival was 120 min (IQR = 90 to 180) and 142 min (IQR = 112 to 180) between ED arrival and the performance of head CT-scan. Time of the mTBI event was unknown for 117 patients (32.1%). The history of the injury and the clinical findings at ED are displayed in Table 2 with univariate analysis according to the presence of intracranial lesions.

**Table 2:** Injury history and clinical findings at the ED with univariate analysis according to the presence of intracranial lesion

	Population (***N*** = 365)	Intracranial lesion (***N*** = 26)	No intracranial lesion (***N*** = 339)	***p***-value
Presence of witness	101 (27.7)	7 (26.9)	94 (27.7)	0.92
**Mechanisms of injury (n, %)**				
Ground-level fall	335 (91.8)	25 (96.1)	310 (92.3)	0.40
Fall from > 1 m or 5 stairs	14 (3.8)	1 (3.9)	13 (3.6)	0.65
*Mechanical fall*	167 (47.9)	9 (42.3)	158 (50.7)	0.41
*Unknow*	142 (40.7)	10 (38.5)	132 (38.9)	0.96
*Faintness or vertigo*	27 (7.7)	3 (11.5)	24 (7.1)	0.30
*Syncope*	13 (3.7)	2 (7.7)	11 (2.3)	0.26
Motor vehicle accident	7 (1.9)	0 (0.0)	7 (1.8)	0.59
Unknown	6 (1.6)	0 (0.0)	6 (1.6)	0.64
Head striking by an object	3 (0.9)	0 (0.0)	3 (0.7)	0.80
**Activity after falling (n, %)**				
Getting up with assistance	203 (55.6)	20 (76.9)	183 (54.0)	0.02
Staying on the floor for > 1 h	108 (29.6)	4 (15.4)	104 (30.7)	0.1
Getting up alone	54 (14.8)	2 (7.7)	52 (15.3)	0.29
**Symptoms after injury (n, %)**				
Unknown	49 (13.5)	2 (7.7)	47 (14.0)	0.37
Amnesia	48 (13.2)	1 (3.8)	47 (13.9)	0.1
Headaches	43 (11.8)	11 (42.3)	32 (9.4)	< 0.001
Loss of consciousness	26 (7.1)	1 (3.8)	25 (7.4)	0.50
Vomiting	11 (3.0)	3 (11.5)	8 (2.4)	0.004
Seizure	2 (0.6)	0 (0.0)	2 (0.6)	0.9
**Clinical examination**				
Glasgow score:				
*15*	326 (89.3)	19 (73.1)	307 (90.6)	0.005
*14*	36 (9.9)	7 (26.9)	29 (8.6)	0.002
*13*	3 (0.82)	0 (0)	3 (0.9)	0.80
Base skull fracture sign	13 (3.6)	3 (11.5)	10 (2.9)	0.06
Alcohol intoxication (n, %)	7 (1.9)	1 (3.9)	6 (1.8)	0.41
Focal neurological sign	3 (0.8)	3 (11.5)	0 (0.0)	< 0.001
**Cutaneous injury (n, %)**				
Wound requiring suture	131 (35.9)	13 (50.0)	118 (34.8)	0.12
Hematoma	117 (32.0)	8 (30.8)	109 (32.2)	0.15
Cutaneous abrasion	78 (21.4)	5 (20.0)	73 (21.5)	0.69
No cutaneous lesion	39 (10.7)	0 (0.0)	40 (11.8)	0.04
Cutaneous impact location (n, %)				
*Frontal*	151 (41.4)	10 (38.5)	141 (41.4)	0.76
*Facial*	86 (23.4)	1 (3.8)	85 (25.0)	0.01
*Temporoparietal*	52 (14.3)	8 (30.8)	44 (13.0)	0.01
*No cutaneous impact*	39 (10.7)	0 (0)	39 (11.8)	0.04
*Occipital*	37 (10.1)	7 (26.9)	30 (8.8)	0.003
**Associated traumatic and/or medical emergency (n, %)**				
Isolated Mild Traumatic Brain Injury	141 (38.6)	11 (42.3)	130 (35.6)	0.67
Traumatic injury	71 (19.5)	5 (19.2)	66 (19.5)	0.97
*Spine or limb fracture*	39 (10.7)	4 (15.4)	35 (10.3)	0.30
*Facial bones fracture*	9 (2.5)	1 (3.9)	8 (2.4)	0.19
Medical emergency	58 (8.8)	2 (7.7)	56 (16.5)	0.24

**Fig. 1:**
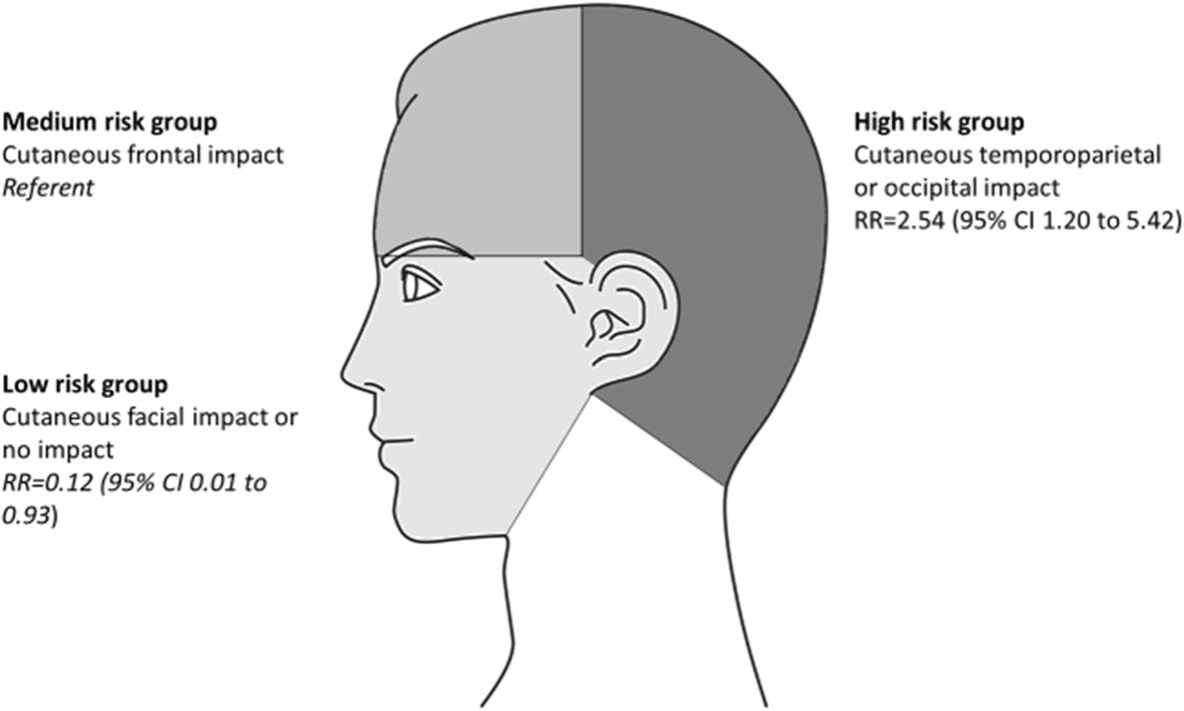
Univariate relative risk of intracranial injury according to the cutaneous impact location

Of the 365 patients included, 26 (7.1%) had intracranial lesion. Compared with cutaneous frontal impact, the relative risk of intracranial lesion was 2.54 (95% CI 1.20 to 5.42) for patients with temporoparietal or occipital impact and 0.12 (95% CI 0.01 to 0.93) for patients with cutaneous facial impact or no cutaneous impact. Relative risk of intracranial lesions according to the cutaneous impact location are presented on Fig. 1. There was no statistical increase in risk of intracranial lesion in patients receiving antiplatelets (RR = 1.43; 95% CI 0.68 to 2.99) or anticoagulants (RR = 0.98; 95% CI 0.45 to 2.14). The univariate analysis of relative risk for intracranial lesions are displayed on Table 3.

**Table 3:** Univariate analysis of relative risk for intracranial lesion after mTBI among patients aged of 65 years old and over presenting to the ED

	Relative risk	95% CI	***p***-value
**Sex**			
Male	1.00		
Female	2.76	0.98–7.84	0.043
**Getting up with assistance**			
Getting up alone	1.00		
Getting up with assistance	2.66	1.09–6.47	0.023
**Cutaneous impact location**			
Frontal	1.00		
Temporoparietal or Occipital	2.54	1.19–5.42	0.012
Facial or no cutaneous impact	0.12	0.01–0.93	0.014
**Headaches**			
No headaches	1.00		
Headaches	5.49	2.70–11.17	< 0.001
**Vomiting**			
No vomiting	1.00		
Vomiting	4.19	1.48–11.91	0.008
**Focal neurological sign**			
No focal neurological sign	1.00		
Focal neurological sign	15.74	10.59–23.38	< 0.001
**Glasgow**			
15	1.00		
14	3.34	1.51–7.39	0.002

Among the 26 patients with intracranial lesions, 3 (11.5%) had a focal deficit: one presented aphasia (3.8%), one homonymous hemianopsia (3.8%), and one hemiparesis (3.8%). Thirteen patients (50%) showed a subdural hematoma, 9 (34.6%) had subarachnoid hemorrhage, and 4 (15.4%) an intraparenchymal hematoma. No patient required neurosurgical intervention. Eighteen patients (69.2%) with intracranial lesion were hospitalized and 2 (7.7%) were transferred to the intensive care unit. Warfarin effects were reversed by prothrombin complex concentrate and vitamin K in 5 patients (19.2%). One patient treated with antivitamin K had an extensive intraparenchymal hematoma despite reversion and died 2 days after admission.

Eighty-nine patients presented cortical subcortical atrophy on head CT-scans; 8 of them (9.0%) had an intracranial lesion (*p* = 0.43).

Overall, 106 patients (29.0%) were hospitalized and 141 (38.6%) had an isolated mTBI.

Patients’ characteristics, injury history, clinical findings and outcomes according to cutaneous impact location according to the cutaneous impact location are presented in the Table 4.

**Table 4:** Patients’ characteristics, injury history, clinical findings and outcomes according to the cutaneous impact location with univariate analysis

	Low risk group (***N*** = 125)	Medium risk group (***N*** = 151)	High risk group (***N*** = 89)	***P*** value
Age (years, SD)	87.8 (7.9)	85.7 (8.3)	85.9 (9.3)	0.28
Clinical Frailty scale (mean, SD)	4.6 (1.6)	4.7 (1.7)	4.4 (1.6)	0.98
**Residence (n, %)**				0.22
Nursing home	57 (45.6)	55 (36.4)	32 (35.9)	
Home	68 (54.4)	96 (63.6)	57 (64.1)	
**Medication (n, %)**				
Antiplatelet	46 (28.8)	58 (38.4)	33 (37.1)	0.96
Anticoagulant	47 (37.6)	50 (33.1)	31 (34.8)	0.74
Psychoactive drugs	52 (41.6)	57 (37.8)	29 (32.6)	0.41
**Ground-level fall (n, %)**				0.31
Mechanical fall	53 (42.4)	73 (48.3)	38 (42.7)	
Unknow	49 (39.2)	54 (35.8)	30 (33.7)	
Faintness or vertigo	9 (7.2)	6 (4.0)	11 (12.4)	
Syncope	3 (2.4)	6 (4.0)	3 (3.4)	
**Symptoms after injury (n, %)**				
Unknown	18 (14.4)	22 (14.6)	6 (6.7)	0.16
Amnesia	20 (16.0)	15 (9.9)	13 (14.6)	0.29
Headache	13 (10.4)	12 (7.9)	10 (11.2)	0.01
Loss of consciousness	12 (9.6)	6 (3.9)	8 (9.0)	0.14
Vomiting	4 (3.2)	1 (0.7)	6 (6.7)	0.03
Seizure	0	1 (0.7)	0	0.53
**Clinical examination (n, %)**				
Glasgow score				0.1
15	112 (89.6)	132 (87.4)	82 (92.1)	
14	10 (8.0)	19 (12.6)	7 (7.9)	
13	3 (2.4)	0	0	
Wound requiring suture	28 (22.4)	68 (45.0)	35 (39.3)	< 0.001
Hematoma	38 (30.4)	49 (32.5	32 (35.9)	0.7
Cutaneous abrasion	21 (18.8)	34 (25.0)	22 (26.2)	0.38
**Intracranial lesion (n, %)**				0.16
Subdural hematoma	1 (0.8)	2 (1.3)	10 (11.2)	
Subarachnoid hemorrhage	0	6 (4.0)	3 (3.4)	
Intraparenchymal hematoma	0	2 (1.3)	2 (2.2)	
**Outcome (n, %)**				0.51
Hospitalization	33 (26.4)	42 (27.8)	29 (32.6)	
ICU	1 (0.8)	1 (0.7)	0	

## Discussion

Our mTBI-related intracranial lesion prevalence of 7.2% was consistent with recent studies performed in the elderly [9, 13]. This lower prevalence found in EDs recent studies may have several causes. Firstly, current guidelines recommend a head CT-scan in most patients over 65 years old after mTBI. Thus, this large indication may decrease intracranial lesion prevalence among this population. Secondly, according to the mTBI definition of 1993, injury mechanisms were not only direct head strikes but also acceleration/deceleration movements without any direct external trauma of the head [14]. In accordance with literature our findings showed that ground-level fall was the most common cause of mTBI [1, 13]. Therefore, the amount of mTBI kinetics involved in ground level fall in the elderly is less than in younger populations (ie: falls from over one meter or motor vehicle accidents). Thus, ground-level fall, especially without head impact, may not have enough kinetics to induce intracranial lesion in the elderly.

Compared with cutaneous frontal impact, temporoparietal and occipital impact had an intracranial lesion relative risk of 2.54 (95% CI 1.19 to 5.42). To our knowledge, the cutaneous impact location in the context of mTBI in elderly has not been assessed. Several hypotheses could explain these results. The musculature reduction of the trunk and neck, to which ageing of the individual contributes, could increase the force of temporoparietal or occipital impact in the event of ground-level fall [4, 15]. Conversely, in case of frontal impact, some of this kinetic energy may be reduced by postural adaptation reflexes of the upper limbs. In addition, frontal impact may have less risk of intracranial lesion due to the fact the frontal bone is thicker than temporal, parietal and occipital bones, [16]. Moreover, in our study and according to literature, 50% of intracranial injuries were subdural hemorrhages [4, 13]. The pathophysiology of this hemorrhage as Miller JD et al. reminds us, involves a direct trauma with low kinetic energy able to affect the venous network of the dura mater and the arachnoid [17]. Furthermore, several hypotheses involving the vulnerability of vascular tissue and age-related white matter alterations have been put forward to explain the susceptibility of old patients to hemorrhage subsequent to a direct trauma with low kinetic energy [18, 19]. The cutaneous impact location in a context of mild traumatic brain injury has already been studied among children. In a recent large study of 3866 children younger than 17 years old, Burns EC et al., [20] showed that temporal/parietal and occipital impact location had significantly greater odds of intracranial lesions than other impact locations. Furthermore, these odds were greatest in children aged from 0 to 6 months. The mechanisms involved in youngest children and frailty elderly might be the same: despite the low kinetic energy, the absence of postural adaptation reflexes of the upper limbs may trigger intracranial lesions especially in case of temporal/parietal and occipital impact. Taken together, these findings could explain why patients with cutaneous temporoparietal or occipital impact had a higher risk of intracranial lesion than those with cutaneous frontal impact. Thus, to better apprehend the relevance of head CT-scan in the context of ground-level fall with mTBI among the elderly, risk groups of intracranial lesion may be identified regarding the cutaneous impact location as follow: low risk groups (with facial or no cutaneous impact), medium risk (cutaneous frontal impact) and high risk (cutaneous temporoparietal and occipital impact). This objective sign may help us to better assess the risk of intracranial lesions and better target head CT-scan indication.

In spite of the prospective nature of our study, it was not possible to determine the fall cause in more than one third of the cases. Timler et al. showed in their retrospective study that the mTBI mechanism was not identified in 23.6% of the cases [13]. Furthermore, in our study, symptoms after mTBI were unknown in 13.5% of the cases. Not only the symptom sensitivity for intracranial lesion diagnosis are low, but the occurrence of those are also often unknown by the physician in charge of the patient [7, 13, 21]. These results strengthen the hypothesis of Papa et al. suggesting that the term mTBI is sometimes misused in the elderly [7]. However, clinical findings as headaches (RR = 5.49; 95% CI 2.70–11.17), vomiting and focal neurological signs (RR = 15.74; 95% CI 10.59–23.37) were significantly associated with the development of intracranial lesions. Regarding the Glasgow score, a score of 14 was significantly associated with intracranial lesion (RR = 3.34; 95% CI 1.51–7.39). Literature shows divergent findings; on the one hand, some studies discredited Glasgow score in elderly by showing less sensitive than in younger patients to detect intracranial lesion [22]. On the other hand, some studies suggested that a Glasgow score limit of 14 instead of 13 may improve sensitivity to predict poor outcome in elderly [23].

Ongoing treatments with antiplatelets or anticoagulants were not associated with a significant increase in the risk of intracranial lesion. Recent studies suggest that the use of antiplatelet alone does not increase the risk of mTBI-related intracranial lesion [24]. About anticoagulant, especially in ground-level fall context, recent studies suggest an increasing risk of intracranial lesion but not a worsen morbidity or mortality [25, 26]. These results should be analyzed carefully, despite the absence of multivariate analysis performed in our study due to the small sample size of it. Moreover, even if ICL prevalence was similar we still notice a higher prevalence in the Aspirin group with 9.8% (11/112) and 8.3% in the Warfarin group with 8.3% (5/60) than the overall population (7.2%, 26/365).

Contrary to our initial hypothesis, patients’ frailty assessed by the Clinical Frailty Scale was not associated with a significant increase in the risk of developing intracranial lesion. However, the frailty measured by this scale was high in our population (mean 4.6/7; SD 1.6). This finding is consistent with literature suggesting that frailty is associated with an high incidence of mTBI [15]. Nonetheless, getting up with assistance was associated with great risk of intracranial lesion (RR = 2.66; 95% IC 1.09–6.47)**.** This may reflect declined functional abilities in these patients who might have impaired postural adaptive reflexes.

Additionally, we identified the same risk factors for intracranial lesion classically identified in older patients such as female (RR = 2.76; 95% CI 0.98–7.83) [21]. The age population average was high. Due to this Fournier et al. suggest increasing the age limit from 65 to 75 years-old. The fact of adjusting the age limit to 75 year-old in the Canadian head CT rule may reduce head CT-scans performed of 25% without any intracranial lesion missed [27]. In their retrospective cohort, Riccardi et al. even suggested to increase this limit to 80 years old [9].

Hospitalization rate in our cohort was low (29%). This is consistent with literature and reinforces recent hypotheses of potentially avoidable and/or inappropriate use of emergency services, particularly for the NH residents counting for 39.5% of our cohort [13]. The following term: ‘potentially avoidable and/or inappropriate use of emergency services’ has no consensual definition, but may represent almost half of the transfers to the ED from nursing homes [28]. Better target intracranial lesions risk after mTBI among this population may reduce the need for ED visits. Our study further strengthens this theory since 38.7% of the patients admitted to the ED had an isolated mTBI, without wounds requiring suture or any medical and/or trauma emergency care.

The strength of our prospective study relies on the anamnesis accuracy and clinical findings in the ED since it was a real-life observational study. This prospective inclusion is a true reflection of the semiological survey conducted by ED practitioners. The main limitation of our study was the population’s size. Its main objective was to establish the prevalence of intracranial lesion and it may lack power to identify certain risk factors. Furthermore, the number of cases was not sufficient to conduct a multivariate analysis. In addition, there was no patient follow-up while according to literature, there might be an increased risk of delayed hemorrhage especially in patients treated with anticoagulants [29]. Moreover, the readmission rate to hospital within 1 month after an ED visit for mTBI-related intracranial lesions might be high, particularly for the elderly with fall-related mTBI [30, 31].

## Conclusions

To sum up, the prevalence of mTBI-related intracranial lesion in elderly patients admitted to the ED was similar to younger patients. This finding might be partly explained by the low kinetic energy and the pathophysiology of hemorrhage in the context of ground-level fall. These results suggest that the cutaneous impact location may help to identify risk for mTBI-related intracranial lesions in older patients. A prospective, multicenter ED study would be useful to confirm these potential risk factors.

## Data Availability

The datasets generated during the current study are available from the corresponding author on reasonable request.

## Abbreviations

CT-scan: Computed Tomography scan
ED: Emergency Department
NH: Nursing Home
mTBI: Mild Traumatic Brain Injury
